# Integrative High-Throughput Screening and Microscopic Evidence Implicates Microsporidia as a Potential Pathogen of “Pus Crab” in the Mud Crab (*Scylla paramamosain*)

**DOI:** 10.3390/ani15233463

**Published:** 2025-12-01

**Authors:** Lanfei Xiao, Yongjun Liang, Shuangli Hao, Kun Wu

**Affiliations:** 1 Nansha-South China Agricultural University Fishery Research Institute, Guangzhou 511464, China; 2 College of Marine Sciences, South China Agricultural University, Guangzhou 510642, China

**Keywords:** *Scylla paramamosain*, pus crab, pathological, metagenomic, microsporidia

## Abstract

The mud crab (*Scylla paramamosain*), an economically important crustacean aquaculture species in southern China, is susceptible to infections due to its immune system lacking acquired immunity. An emergent disease locally termed “pus crab” has caused severe muscle lesions in pond-farmed crabs, but its etiology remained unclear. Here, we applied an integrated approach, histopathology, electron microscopy, metagenomic sequencing, and experimental infection to identify the pathogen of “pus crab”. Histological staining (H&E, Wright–Giemsa, and Masson) revealed muscle fiber dissolution, disordered fiber arrangement, and abundant interstitial spore-like bodies. Scanning electron microscopy (SEM) and transmission electron microscopy (TEM) confirmed intracellular spore morphology consistent with microsporidia. Metagenomic profiling showed a pronounced shift in the muscle microbiome, with a marked increase in microsporidian taxa at the genus level and a concurrent decline in bacterial relative abundance. Functional annotation indicated enrichment of pathways related to protein processing, ribosome biogenesis, glycosylation, and the ubiquitin–proteasome system. Isolation of spores from diseased muscle and subsequent injection into healthy crabs reproduced wild-like clinical signs and histopathology, confirming infectivity and implicating microsporidia as the likely etiological agents of “pus crab”. These findings establish a multidisciplinary framework for pathogen identification in aquaculture and provide candidate molecular and biochemical markers for early diagnosis and management.

## Simple Summary

The mud crab (*Scylla paramamosain*) is an economically important aquaculture species in southern China, but its lack of acquired immunity makes it highly susceptible to diseases. In recent years, a locally termed “pus crab” disease has been spreading in farming ponds, primarily affecting muscle tissue and causing significant economic losses. To identify the potential pathogen, we conducted omics-based identification and ultrastructural observations. The results confirmed abundant microsporidia in the muscle tissue of infected crabs, and experimental infection with isolated microsporidia successfully reproduced the disease phenotype in healthy crabs. Therefore, we speculate that microsporidia are the potential causative agent of “pus crab” disease. Our research provides scientific support for the prevention and control of aquatic diseases, contributing to sustainable aquaculture development and food safety.

## 1. Introduction

The mud crab (*Scylla paramamosain*), a globally significant economic crab species, holds a central position in the aquaculture industries of Southeast Asia and southeastern China due to its delicious meat, rich nutritional profile, and strong adaptability, with annual production exceeding 150,000 tons [1]. However, with the rapid expansion and intensification of farming practices, disease outbreaks have become a major constraint on sustainable development. Notable pathogens include white spot syndrome virus [2] and *Hematodinium* spp. [3]. In addition, a condition locally referred to as “pus crab” has emerged over the past decade in Guangdong, Hainan, and other regions. Early infections present no obvious symptoms, but as the disease progresses, affected crabs die and sink to the pond bottom (field observation), causing severe economic losses. Farmers have traditionally relied on antibiotics (e.g., enrofloxacin and florfenicol), disinfectants (e.g., povidone-iodine), and Chinese herbal preparations for disease management. Yet, these interventions have yielded limited success and are often accompanied by adverse consequences, including drug resistance, environmental pollution, and chemical residues in seafood products [4]. The fundamental challenge lies in the unresolved etiology of “pus crab” disease. Conventional approaches—morphological observation and microbial isolation—have failed to identify definitive pathogens from affected tissues. A few studies have speculated on possible associations with parasites (e.g., *Hematodinium* spp.) [5] or fungi (e.g., *Metschnikowia bicuspidata*) [6], but conclusive evidence remains lacking. This persistent gap in pathogen identification has impeded the development of effective prevention and control strategies, making it the foremost scientific challenge in current research on *S. paramamosain* health research.

Traditional pathogen identification primarily relies on microscopic observation (e.g., Gram staining and tissue sectioning) [7], pathogen isolation and culture (e.g., bacterial growth characteristics on TCBS agar) [8], etc. However, these methods exhibit significant limitations when addressing complex mixed infections or unknown pathogens. The emergence of high-throughput sequencing technologies, particularly metagenomics and metatranscriptomics, has provided powerful alternatives for overcoming these challenges. Metagenomics encompasses the total microbial genetic material within an environment, including genomes of both culturable and non-culturable microorganisms, which is widely applied to investigate microbial community composition, functional potential, and interactions, and is now regarded as the gold standard for microbiome research [9]. Depending on the target organisms, metagenomic studies are often categorized into viral and bacterial metagenomics: the former emphasizes viral community structure and function, while the latter focuses on bacterial diversity and metabolic capacity [10]. Beyond taxonomic profiling, metagenomic analyses provide valuable insights into virulence factors of pathogenic bacteria, metabolic pathway, and gene prediction [11,12]. Its wide application also highlights potential for exploring host biological responses such as immunity, inflammation, and stress regulation.

Microsporidia are a diverse group of unicellular obligate intracellular parasites that were first discovered in silkworms over 150 years ago [13]. Initially recognized as protozoa, they are now classified as a basal branch or sister group of fungi [14] and can infect both vertebrates and invertebrates [1]. In 1977, Microsporidia was elevated to phylum level from class or order level, and until 1998, the term Microsporidia was formally adopted to replace the earlier designation Microspora [15]. The life cycle of microsporidia is distinctive, consisting of the infective or environmental phase, the proliferative phase, and the sporogonic or spore-forming phase [16]. Of these, only the spore stage can survive in vitro, and the spores have diverse morphologies, including oval, round, and occasionally spindle-shaped forms [17]. Nearly 50 genera of microsporidia are known to infect crustaceans, with the hepatopancreas serving as the main parasitic site, followed by muscle [18]. Accurate identification remains challenging due to their vast diversity, and preliminary classification still relies largely on molecular markers such as Small Subunit (*SSU*) rRNA, Large Subunit (*LSU*) rRNA, the Internal Transcribed Spacer (ITS) region, etc. [19]. Furthermore, microsporidia infection can cause a range of functional disorders in the host, creating favorable conditions for secondary infections by other pathogens [20].

To address this knowledge gap, we divided collected mud crabs into two groups for muscle histopathology and electron microscopy, identified potential pathogens using metagenomic approaches, and subsequently verified pathogenicity through in vivo injection experiments.

## 2. Materials and Methods

### 2.1. Sample Collection

**Figure 1:**
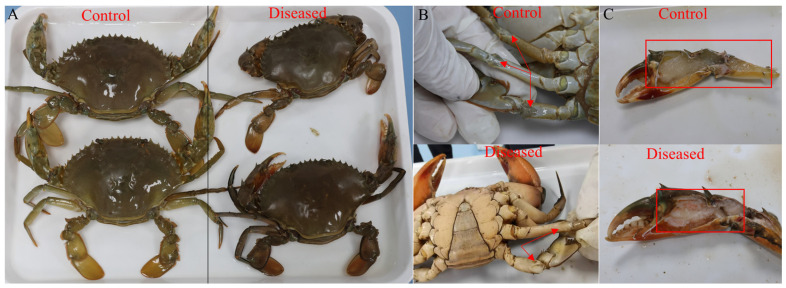
Comparison of appearance (**A**), joints (**B**), and skeletal muscle (**C**) between control and diseased crabs. The red arrows or frames indicate the milky joints and slightly liquefied skeletal muscle of the diseased crab.

The diseased and control mud crabs were collected from the same pond in Jiangmen (Guangdong, China), which had the following water quality parameters: a pH of 7.08 ± 0.14, dissolved oxygen ≥ 6.31, a salinity of 1.96‰ ± 0.03, and a temperature of 28.22 °C ± 0.26. The affected crabs exhibited obvious symptoms, including appearing dull in color, milky white joints, and slightly liquefied skeletal muscle (Figure 1). Samples stored in an ice box were quickly transported to the laboratory of Nansha-South China Agricultural University Fishery Research Institute, Guangzhou. Upon arrival, muscle samples were dissected using scissors and tweezers, put in 15 mL tubes, quickly frozen immediately in liquid nitrogen, and then stored at −80 °C. The same segment of muscle was sampled and stored in 4% paraformaldehyde solution (Biosharp, Hefei, China) for hematoxylin–eosin (H&E), Wright–Giemsa, and Masson staining analysis.

### 2.2. Hematoxylin–Eosin, Wright–Giemsa, and Masson Staining Analysis

According to our previous study and standard histological procedures [7], samples were fixed in 4% paraformaldehyde, dehydrated in a series of alcohol solutions, and infiltrated with xylene. Subsequently, the samples were stained with H&E, Wright–Giemsa, or Masson staining and processed on histological slides. The dyeing solution is provided by Solarbio (Beijing, China). Neither the producer nor the analyst knew the specific grouping.

### 2.3. Electron Microscope Analysis

The muscles were rinsed clean, fixed with 2.5% glutaraldehyde solution (Macklin, Shanghai, China), and stored at 4 °C in the dark. The fixed samples were subjected to gradient dehydration and drying, and then gold-coated for scanning electron microscopy (SEM, Hitachi, Hitachi, Japan) observation. For transmission electron microscopy (TEM, Hitachi, Hitachi, Japan), the samples were fixed with osmium phosphate for a second time, embedded after dehydration, and transferred to copper grids for lead staining and observation after ultrathin sections.

### 2.4. DNA Extraction and Illumina High-Throughput Sequencing

Three diseased and control crabs were randomly selected, respectively, with approximately 300 mg of skeletal muscle from chelipeds used for DNA extraction. DNA was extracted using the E.Z.N.A.^®^ DNAKit (Omega Bio-tek, Norcross, GA, USA) according to the manufacturer’s instructions. Qualified genomic DNA was firstly fragmented by sonication to a size of 450 bp and then end-repaired, A-tailed, and adaptor-ligated using the NEBNext^®^ ΜLtra™ DNA Library Prep Kit (NEB, Beverly, MA, USA) for Illumina according to the preparation protocol, and DNA fragments were enriched by PCR. Genome sequencing was performed on the Illumina Novaseq 6000 sequencer (Illumina, San Diego, CA, USA) using pair-end technology (PE 150). Clean reads removed host genome contamination and low-quality data for further analysis.

### 2.5. Metagenomic De Novo Assembly, Gene Prediction, and Functional Annotation

After quality control, sequencing data from each sample were assembled into contigs using MegaHit v1.0 software. Open reading frames (ORFs) were predicted from the assembled sequences, and non-redundant gene sets were generated using CD-HIT v4.8.1 software. For each cluster, the longest sequence was designated as the representative sequence.

Functional annotation of the predicted genes was performed using multiple databases, including the Kyoto Encyclopedia of Genes and Genomes (KEGG), Evolutionary Genealogy of Genes: Non-Supervised Orthologous Groups (eggNOG), and Carbohydrate Active Enzymes Database (CAZy) to infer metabolic pathways, orthologous relationships, and carbohydrate-active enzyme functions.

### 2.6. Isolation and Regression Infection of Microsporidia

Muscle-derived microsporidia spores were isolated from tissue following a modified version of the method of isolating *Enterocytozoon hepatopenaei* (EHP) [21]. In brief, the diseased crab muscle was added to 1 × phosphate-buffered saline (PBS, Biosharp, Hefei, China) for grinding, the filtrate was collected after filtering with medical gauze and centrifuged at 8000 rpm for 5 min, and the supernatant was discarded; after repeating three times, the precipitate was resuspended in PBS and carefully added to the upper layer of the gradient Percoll^®^ solution (Solarbio, Beijing, China), centrifuged at 17,800 rpm at 15 °C for 30 min, and the purified spores were collected. Subsequently, the DNA of the purified spores was extracted and identified using microsporidium general primers V1f (5′-CACCAGGTTGATTCTGCC-3′) and 1492r (5′-GGTTACCTTGTTACGACTT-3′) [22,23]. The PCR reaction was carried out in a total volume of 50 μL, containing 45 μL of 1.1 × S4 Fidelity PCR Mix (Genesand Biotech Co., Ltd., Beijing, China), 2 μL of forward primer, 2 μL of reverse primer, and 1 μL of DNA template. The PCR reactions were performed with the following program: 98 °C for 30 s; 98 °C for 10 s, 50 °C for 15 s, and 72 °C for 15 s (35 cycles), followed by 72 °C for 5 min. Primers were obtained from Sangon, Inc. (Shanghai, China). Sanger sequencing was performed on the PCR products, and the obtained sequences were compared with Blast (https://blast.ncbi.nlm.nih.gov/Blast.cgi (accessed on 20 October 2025.)) to identify possible species.

According to the research of EHP, the number of purified spores was adjusted to 4 × 10^6^ CFU/mL [24]. Forty healthy mud crabs (~45 g) were divided into two groups; the injection group (20 crabs) was administered a foot joint membrane injection of 0.1 mL of spores, while the PBS group received an injection of 0.1 mL of PBS. The crabs were maintained in natural seawater, and clinical signs were monitored until characteristic muscle phenotypes became evident.

### 2.7. Statistical Analysis

The *t*-test was used to test for statistical differences between the two groups. All statistical analyses were performed using SPSS 17.0 software (SPSS Inc., Chicago, IL, USA). Data were presented as means ± standard deviation (SD), and *p* < 0.05 was considered statistically significant. All graphics were prepared using GraphPad Prism 8.0 software.

## 3. Results

### 3.1. Histopathological Changes of Muscle

**Figure 2:**
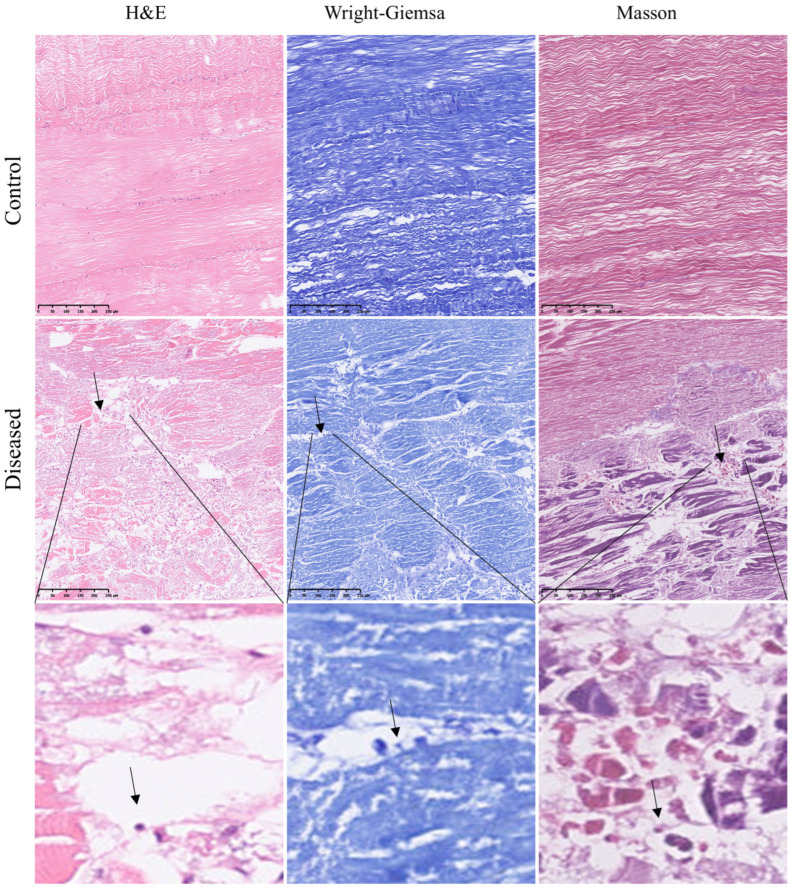
H&E, Wright–Giemsa, and Masson staining of control and diseased crab muscle. The black arrows indicate potential spores.

**Figure 3:**
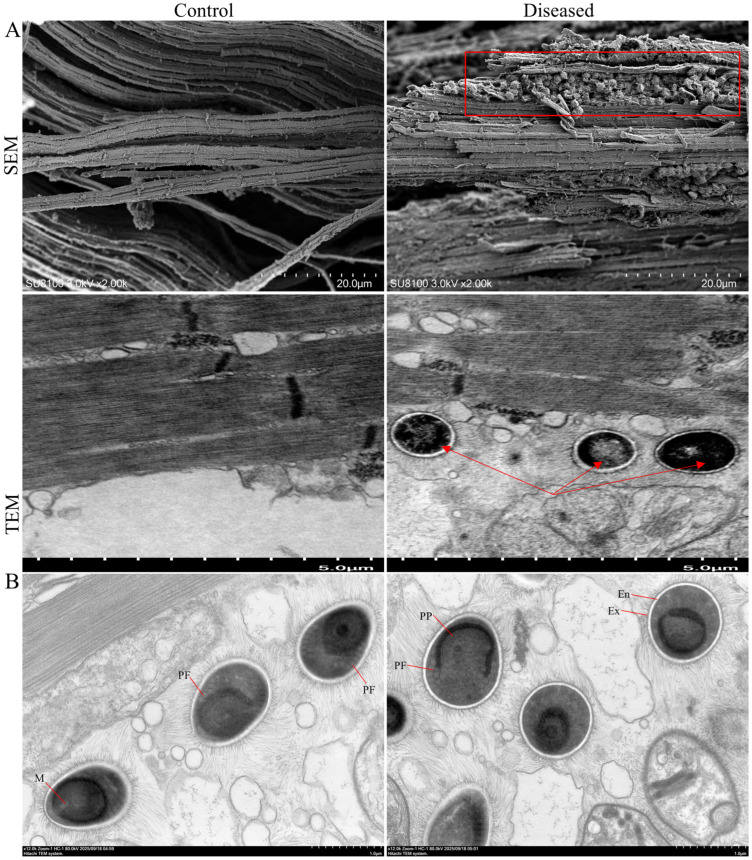
SEM and TEM of control and diseased crab muscle (**A**). The red frame and arrows indicate spores. Spore structure (**B**). Ex—exospore; En—endospore; PP—lamellar polaroplast; PF—polar filaments; M—manubrium or straight portion.

Histological structures of control and diseased crab muscle stained with H&E, Wright–Giemsa, and Masson staining are shown in Figure 2. Compared with the control crabs, diseased crab muscle exhibited pronounced fibrolysis, with fibers appearing disordered and irregular. Similar pathological alterations were confirmed by SEM images (Figure 3A). In addition, spores in the diseased crab muscle were discovered (Figure 2, black arrow), and their surfaces were rough and protruding, connecting with each other to form a network (red frame); TEM confirmed that the protrusions on the spore surface might be villi (Figure 3A, red arrow). Mature spores are found in muscle and have typical spore structures, such as exospore, endospore, and polar filaments, etc. (Figure 3B).

### 3.2. Metagenomic Data Output

**Figure 4:**
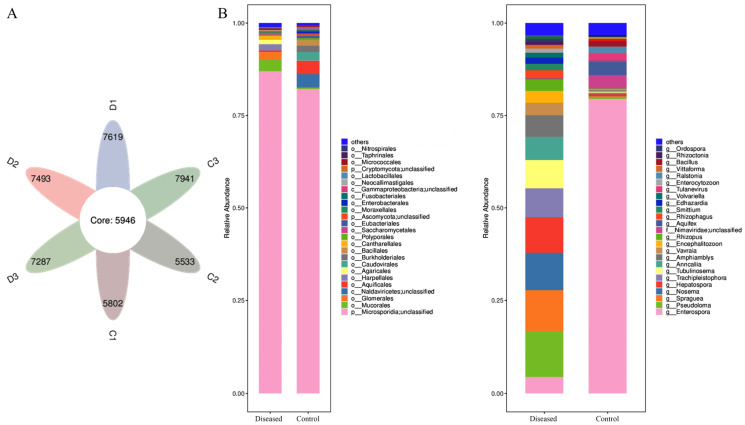
Venn diagram showing the shared genes of muscle microbial composition of mud crabs (**A**). Relative abundance of the muscle microbes in each region at the order and genus level (**B**).

Metagenomic analysis of muscle microbes was performed on control and diseased crabs to identify potential pathogenic microorganisms. After quality control, the number of total reads, host reads, clean reads, and ORF number in the control and diseased group were 41,398,079/41,160,876, 39,271,391/28,848,690, 2,126,688/12,312,187, and 8555/11,485, respectively (Table 1). The markedly reduced proportion of host reads in diseased samples suggests replacement of host tissue by microbial sequences. A total of 18,425 genes were identified across both groups, of which 5946 were shared (Figure 4A). The microbial communities were dominated by bacteria and fungi. At the species level, 86 bacterial taxa were detected in the control group, compared with 54 in the diseased group, whereas fungal taxa increased from 56 in controls to 60 in diseased samples (Table 2). These results indicate a decline in bacterial diversity accompanied by a relative increase in fungal representation in diseased crab muscle.

### 3.3. Muscle Microbial Composition Analysis

The muscle microbial composition of the control and diseased crabs was compared at the order and genus levels (Figure 4B). At the order level, microsporidia have high relative abundance in both control and diseased crabs, while the relative abundance of Mucorales and Glomerales was higher and Naldaviricetes and Aquificales was lower in diseased crabs compared to the control. Meanwhile, at the genus level, it is merely a change in the relative abundance of different microsporidium genera; for instance, *Enterospora* declined, while *Pseudoloma*, *Spraguea*, *Nosema*, *Hepatospora*, etc., increased.

**Figure 5:**
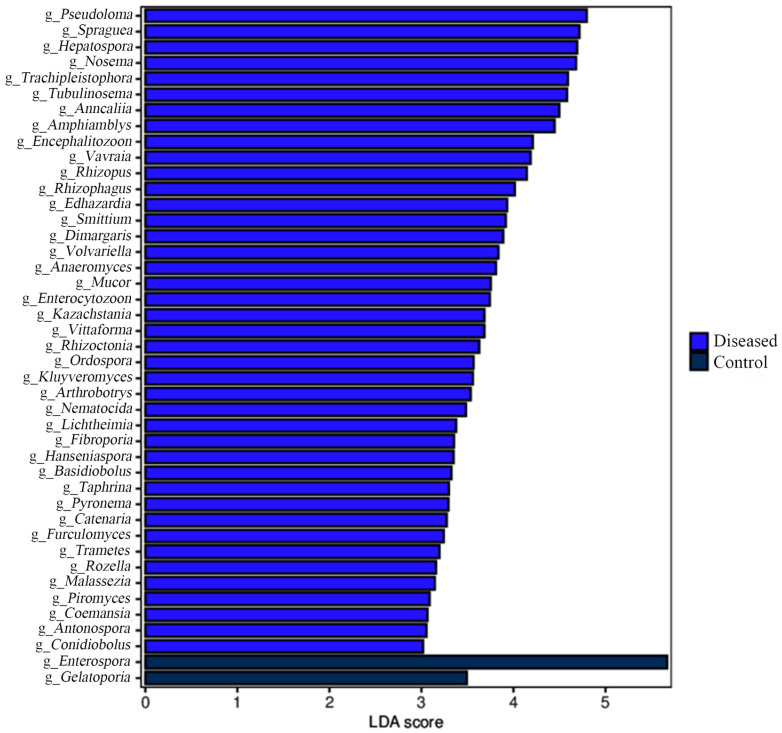
Linear discriminative analysis (LDA) scores showing taxonomic differences were computed at the genus level for the control and diseased crabs. The linear discriminant analysis (LDA) score is represented by the length of the bar.

The microbial signature from the control and diseased crabs was detected using linear discriminant analysis effect size (LEfSe). LEfSe analysis revealed that *Enterospora* was the most abundant in the control crabs, and *Pseudoloma* was the most abundant in the diseased crabs (Figure 5).

### 3.4. Functional Analysis

**Figure 6:**
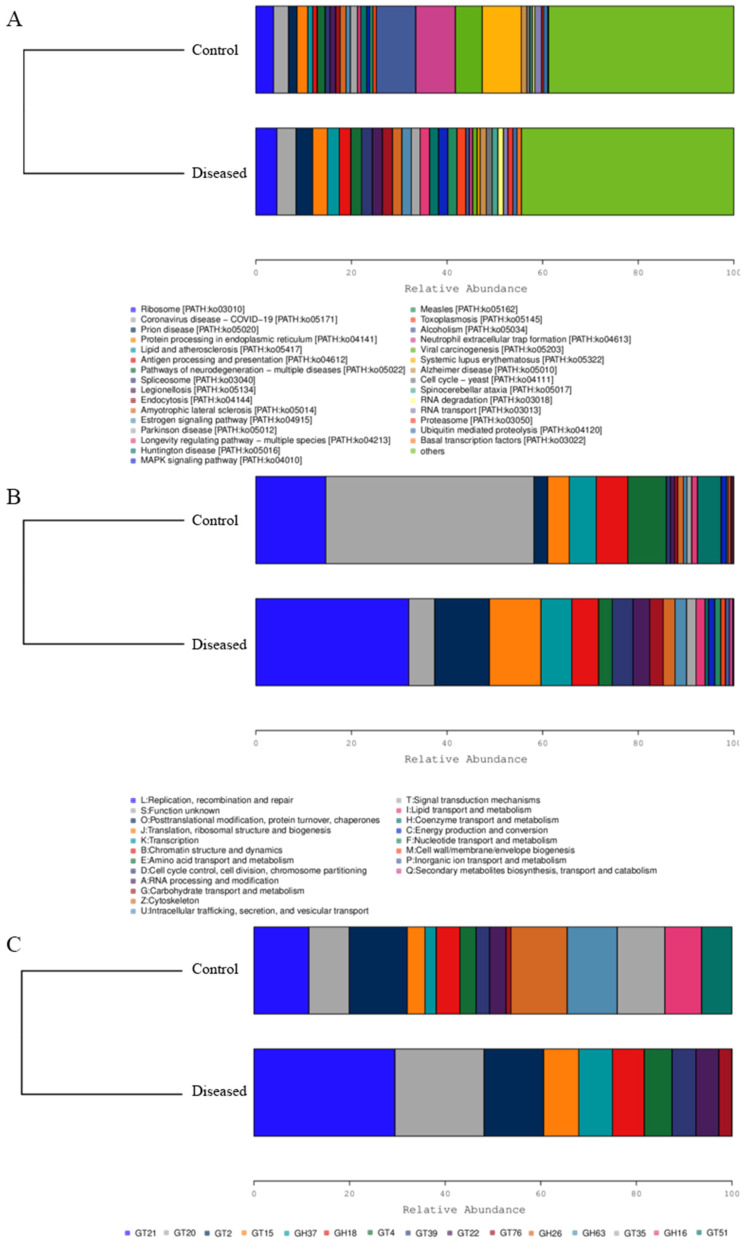
Function relative abundance of muscle microbes in diseased and normal crabs. KEGG (**A**); eggNOG (**B**); CAZy (**C**). GT represents glycosyl transferases, and GH represents glycoside hydrolases.

As shown in Figure 6, the diseased crabs exhibited greater enrichment of several functional pathways. These included ribosome biogenesis and protein processing in the endoplasmic reticulum (Figure 6A), as well as pathways related to replication, recombination and repair, post-translational modification, protein turnover, chaperones, translation, ribosomal structure, and biogenesis (Figure 6B). In addition, the relative abundance of glycosyltransferases in muscle tissue was altered, suggesting that certain compounds may have undergone glycosylation (Figure 6C).

### 3.5. Pathogenicity Study

**Figure 7:**
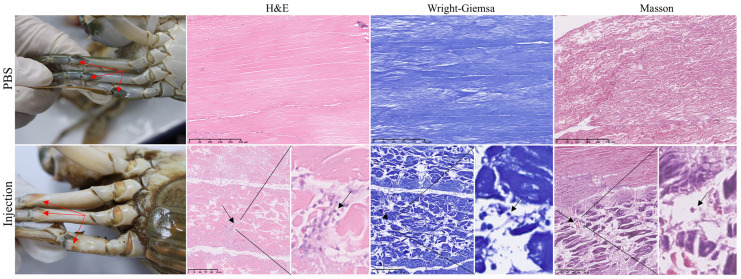
The appearance and pathological changes of crabs injected with microsporidia. The red arrows point to the difference in the color of the joint membrane, and the black arrows point to the main pathological changes.

We conducted PCR on the isolated microsporidium spore DNA. After Sanger sequencing, it was found that it had 99.67% identity with *Ameson portunus*, confirming that the isolated spores might be *A. portunus* (Supplementary Material). Following injection of purified spores, three crabs developed whitening of the joint membranes by Day 5. By Day 11, ten crabs (50% infection rate) exhibited visible symptoms, at which point the observation period was concluded (Figure 7). Histopathological analysis of muscle tissues collected at this stage revealed lesions consistent with those observed in naturally infected crabs, confirming that microsporidia are likely the causative agents of “pus crab” disease. All crabs survived throughout the observation period; however, the experimentally infected group displayed milder symptoms compared with naturally diseased crabs. No abnormalities were observed in the PBS-injected group.

## 4. Discussion

Understanding microbial dynamics in aquaculture systems provides crucial insight into host–microbe interactions under variable environmental conditions and enables targeted microbial management to improve health, productivity, and disease control [25]. These interactions are multifactorial and influenced by water quality, stocking density, and feeding regimes [26]. Clarifying these dynamics supports evidence-based interventions that reduce disease-related losses.

In this study, microsporidia were identified in mud crabs (*S. paramamosain*) by histopathology, ultrastructural analysis, and metagenomics. Experimental infection reproduced clinical signs closely resembling those observed in field cases, strengthening the causal link between microsporidia and the disease and informing potential control strategies. Metagenomic profiling showed that microsporidia accounted for over 75% of the total microbial abundance in muscle at the order level, with genus-level analysis revealing compositional complexity. Microsporidia are obligate intracellular, spore-forming parasites that infect diverse vertebrate and invertebrate hosts [27]. Notably, *Hepatospora* sp. and *Spraguea* sp. were detected in control or diseased muscle, suggesting that mud crabs may carry microsporidia, a finding that contrasts with previous reports [1]. Using specific primers, the potential pathogen of “pus crab” was identified as *A*. *portunus*, which was first discovered in *Portunus trituberculatus* in 2012 [28]. Subsequently, based on SSU rRNA phylogenetic analysis and ultrastructure, it was determined to be a new species of the genus *Ameson* and named *Ameson portunus* n. sp. [18]. In 2023, Zhang first reported that *A. portunus* could infect *S. paramamosain* and that it did not show genetic differentiation from the species identified in swimming crabs [22]. Muscle is the main target organ of *A. portunus* infecting *S. paramamosain*, and in severe infections, it shows the presence of numerous mature spores, which lead to the dissolution of muscle fibers and disrupt the integrity of structure and function [22]. The findings of Zhang et al. are similar to those of this paper, and both identified *A. portunus* from pond mud crabs in Guangdong Province using different preliminary methods. Therefore, we have reason to infer that the “slurry-like syndrome” described by Zhang et al. is the same disease as the “pus crab” we mentioned.

Microsporidia lack oxidative phosphorylation and glycolysis pathways and cannot produce energy by themselves [29]; they steal host energy from multiple organizations by affecting various lipid metabolism pathways, such as those of triglycerides and diglycerides [30]. Muscle is the main source of amino acids to maintain the vigorous metabolism and homeostasis of aquatic crustacean cells, and lesions will undoubtedly reduce the resistance to environmental factors [1,31]. Consistent with this, diseased muscle exhibited dissolution and abundant deeply stained particles, which may be pathogens [1,32]. Further analysis by SEM and TEM subsequently indicated that the pathogen might be spores, as it possesses the typical morphology, such as polar filaments. Polar filaments are key organs for microsporidia to infect host cells [33]. When spores are stimulated by the environment (such as chemical signals from the host cell or changes in osmotic pressure), the polar filaments rapidly evert to form hollow polar tubes, injecting the infectious material (sporoplasm) from the spore into the host cell [34]. Microsporidia are strict intracellular parasites that consume a large amount of nutrients from the host, which in turn leads to a decrease in muscle protein levels. A decline in muscle protein may lead to muscle atrophy and changes in energy supply, thereby affecting oxidative stress and the immune system [35,36].

The ubiquitin–proteasome system (UPS) pathway in the diseased crab muscle was significantly enriched. The UPS plays an important role in maintaining cellular proteostasis and regulating protein functions [37]. Recent studies have shown that the UPS is involved in the host’s immune response to microsporidia infection. On the one hand, microsporidia may evade immune clearance by hijacking the host UPS to degrade host defense-related proteins; for example, some microsporidia secrete effector proteins that induce host protein ubiquitination and degradation by proteasomes, thereby weakening the host’s antiviral or antiparasitic immune signaling pathways (such as NF-κB or interferon response) [38]. On the other hand, host cells may also target and degrade key microsporidian proteins through the UPS, limiting their proliferation. Experiments have shown that proteasome inhibitors (such as MG-132) can significantly inhibit the growth of microsporidia, suggesting that the host UPS may be involved in the degradation of parasite proteins [39]. Furthermore, microsporidia themselves may utilize the UPS to regulate their life cycle. For example, spore germination involves ubiquitination of proteins within the host cytoplasm, but the specific mechanism remains unclear [40]. Taken together, these findings indicate a complex interplay in which microsporidia exploit host metabolic pathways, while the host mounts an oxidative and humoral response that may be insufficient to clear intracellular infection. From an applied perspective, targeting oxidative stress and modulating specific UPS nodes could complement biosecurity measures and targeted diagnostics. Future work should examine UPS component expression at the protein level, map ubiquitination targets during infection, and test whether selective modulation of UPS or antioxidant pathways alters microsporidia replication and host outcome. Thus, this study provides a new perspective for the cognition of “pus crab”, clarifies the potential pathogens, and also provides new methods for the healthy cultivation and disease identification of mud crabs.

## 5. Conclusions

In this study, a comprehensive metagenomics analysis was conducted to investigate the microbial community associated with “pus crab”. The results revealed significant alterations in the muscle microbiota, with microsporidia being the predominant genera at the taxonomic level, and *A. portunus* might be a potential pathogen. However, the complex interactions between these microbes and the host crab remain incompletely understood, highlighting the need for further functional and mechanistic investigations. Collectively, this work underscores the importance of integrating metagenomics with histopathology, electron microscopy, and in vivo infection assays to more accurately define microbial functions and pathogenic mechanisms. These integrated approaches provide valuable insights for future research on the microbiota associated with “pus crab,” paving the way for a deeper understanding of disease mechanisms and the development of effective control strategies in aquaculture.

## Figures and Tables

**Table 1:** Sequencing data production.

Index	Control	Diseased
Total reads	41,398,079 ± 2,284,177.55	41,160,876 ± 3,143,707.62
Host reads	39,271,391 ± 2,147,092.00 ^b^	28,848,690 ± 2,279,256.00 ^a^
Clean reads	2,126,688 ± 149,976.30 ^a^	12,312,187 ± 913,305.30 ^b^
ORF number	8555 ± 1296.61 ^a^	11,485 ± 273.00 ^b^

Data are expressed as mean ± SD; different letters in the same line show a significant difference (*p* < 0.05).

**Table 2:** Microbial composition of mud crab muscle in the control and diseased groups.

Sample	Phylum	Class	Order	Family	Genus	Species
Archaea						
Control	2	4	4	4	4	5
Diseased	5	7	7	7	7	9
Bacteria						
Control	10	18	30	46	69	86
Diseased	12	18	31	40	48	54
Fungi						
Control	8	18	19	35	43	56
Diseased	8	21	22	38	46	60
Virus						
Control	3	3	3	3	6	12
Diseased	4	4	4	4	5	5

## Data Availability

All data are contained within the article.

## Supplementary Materials

The following supporting information can be downloaded at: https://www.mdpi.com/article/10.3390/ani15233463/s1, Figure S1: Blast comparison image; Table S1: PCR product sequence.
