# Supplementary material for: Integrative High-Throughput Screening and Microscopic Evidence Implicates Microsporidia as a Potential Pathogen of “Pus Crab” in the Mud Crab (Scylla paramamosain)
Source: Animals (Basel). 2025 Dec 1;15(23):3463. doi: 10.3390/ani15233463 (PMC12691301; doi:10.3390/ani15233463)
Supplement: Supplementary file 1 [file animals-15-03463-s001.zip › animals-4004799-supplementary final.pdf]

Integrative high-throughput screening and microscopic evi-dence implicates microsporidia as a potential pathogen of "pus crab" in the mud crab (*Scylla paramamosain*)

Lanfei Xiao, Yongjun Liang, Shuangli Hao and Kun Wu

Table S1: PCR product sequence

GAGGTCATGCTAAGATTCGCCTGCAAGTAGTATGTATGTATACACAAGG

CTCAGTATCGAGTATAGCTTTGCTCTCCAAGATGTGATACTTTCAGGAA

ACAGAAAATAAAGCATCTATCTTCTAAAGTCTTTTAGAGGAGAGGAGA

AGAAGCGACTCACCTATCAGTTAGTAGGTATGGTAAGGGCATACCTAG

ACGAAGACGGGTACGGGGAAGGCAACTTCGATTCCGGAGAGGGCGC

ATTTAGAGATGGCGACCAGTTCTAAGGAGTGCAGCAGGCTCGAAACTT

ACCGAATTATAGATTAGAGGTAGTGATGAAACGTTTATATAGAAATACT

GGTAAAGCAAGTATTATCAACTGGAGGGAAAGTCTGGTGCCAGCAGC

CGCGGTAATACCAGCTCCAGGAGCTTCTTCGATATGTTGCGGTTAAAAC

GTCCGTAGTCGCGGCTTGGGACTGACCTGTAATCTATTTGGTCAACAGA

TAGATAGGGGCAGTAGCAAGCTGGAAAAGAGCAATTTGGTGTCAGCT

AATGGTATGGGGAGGGGTGAAGTCTGAGGATCCATGCAGGAGGAGCA

AAGGCGAAAGCACTGACAAAGATTGATTCTGTTGATCAAGGACAGAG

GCTAGAGGATCGAATACGATTAGATACCGTAGTAGTTCTAGCAGTGACC

GATGATGATTTTGCTTATGGCAATAGAGAAATCAAAATAGATCTCCGGG

GGGAGTACATGCGCAAGCAAGAAACTTAAAGAAATTGACGGAAGACT

ACCACAAGGTGTGGATTGTTCGGCTTAATTTGACTCAACGCGGGAAAA

CTTACCAAGCTTATTTATTCAACGAGTATTTATACGAGAGTAAAATGGT

GGTGCATGGCCGTTCCTAACAGATGGAGTGATTTTGTGATTAACTTCCG

TAATCTGTGTAATCTCAGAATAGCTTGTTCGAAAGAACAATTCGAGGC

AAGAACAGGTCAGTGATGTCCTTTGATAGCTTGGGCTGCACGCGCAAT

ACAATGTTTTATGTAGTAAGATATAGATAAAAAATAGAACATGAGGGAT

TGAGGGCTGAAAAGCACTCATGAACACGGAATAGCTAGTAATCGTCA

GTTCAATATACGGCGATGAATATGTCCCTGTTCTTTGTACACACCGCCC

GTCGTTATCGAAGATGGAGTGATTTTTGAGTCAATTATAATGGCGAAGA

GTCATCATTTGCGC

Figure S1: Blast comparison image

	Description	Scientific Name	Max Score	Total Score	Query Cover	E value	Per. Ident	Acc. Len	Accession
☑	Ameson portunus small subunit ribosomal RNA gene, partial sequence	Ameson portunus	2189	2189	98%	0.0	99.67%	3465	OQ378357.1
☑	Ameson portunus small subunit ribosomal RNA gene, partial sequence; internal transcribed spacer, complete seq...	Ameson portunus	2183	2183	98%	0.0	99.58%	3465	OQ417619.1
☑	Ameson portunus isolate JQX201209 small subunit ribosomal RNA gene, partial sequence	Ameson portunus	2174	2174	98%	0.0	99.66%	1194	KC915038.1
